# Compressive Properties and Degradable Behavior of Biodegradable Porous Zinc Fabricated with the Protein Foaming Method

**DOI:** 10.3390/jfb13030151

**Published:** 2022-09-13

**Authors:** Qiqi Ge, Xiaoqian Liu, Aike Qiao, Yongliang Mu

**Affiliations:** 1 School of Metallurgy, Northeastern University, Shenyang 110819, China; 2 Faculty of Environment and Life, Beijing University of Technology, Beijing 100124, China

**Keywords:** medical degradation, porous zinc, protein foaming, elasticity modulus, compressive strength

## Abstract

A new protein foaming–consolidation method for preparing porous zinc was developed using three proteins (egg white protein (EWP), bovine bone collagen protein (BBCP), and fish bone collagen protein (FBCP)) as both consolidating and foaming agents. The preparation route utilized powder mixing and sintering processing, which could be divided into three steps: slurry preparation, low-temperature foaming, and high-temperature sintering. The morphological characteristics of the pore structures revealed that the porous zinc had an interconnected open-cell structure. Compared to the porous zinc prepared with EWP or BBCP, the porous zinc prepared with FBCP possessed the largest average pore size and the highest compressive properties. The porosity of the porous zinc increased with the stirring time, the content of protein and sucrose, and higher sintering temperatures. Moreover, a compression test and immersion test were performed to investigate the stress–strain behavior and corrosion properties of the resulting porous zinc. A fluctuated stress plateau could be found due to the brittle fracture of the porous cells. The porous zinc prepared with FBCP showed the highest compressive strength and elastic modulus. The corrosion rate of the porous zinc obtained through an immersion test in vitro using simulated bodily fluids on the thirty-second day was close to 0.02 mm/year. The corresponding corrosion mechanism of porous zinc was also discussed.

## 1. Introduction

Medically degradable metal can be metabolized and absorbed into the human body, and the degradation products are harmless for it. The demand for such metals has growing over recent decades due to their excellent biocompatibility and biodegradability and their adequate mechanical properties [1,2]. Porous material is a potential material for implantation for bone tissue regeneration and substitution. Voids and holes in porous materials can ensure that fresh fluid is easily sent into porous implantation materials, allowing new bone or vascular tissue to grow into the material [3]. With the proliferation and differentiation of new bone or vascular cells, the medically degradable porous material will gradually disappear [4,5]. In addition, as medically degradable porous materials must also possess adequate mechanical properties for supporting enough strength for surgery and in vivo tissue recovery processes [6,7,8].

Over recent decades, bone implantation materials have been made with glass–ceramic [9], polymer [10], and poly-hydroxyapatite composites [11]. However, the mechanical properties of these implantations cannot be satisfactory for the whole process of implantation and healing [12]. On the other hand, though permanent implantation materials such as tantalum [13], titanium [14], and their alloys have adequate mechanical properties, a stress-shielding influence would emerge due to their mechanical properties far more than it would with natural bone [15]. Therefore, degradable metal materials have become a research focus in recent years.

Currently, the most noticeable medically degradable porous metals include zinc (Zn) [16], iron (Fe) [2], magnesium (Mg), and their alloys [17]. Mg possesses suitable mechanical properties and good biocompatibility [18]; however, it releases hydrogen when it degrades in the body, and the degradation rate is so fast that the integrity of the scaffolding function of the implantation cannot be promised [19,20,21]. Fe possesses a higher elastic modulus, which leads to stress shielding [22]. Furthermore, the corrosion rate of Fe is so slow that it would induce the occurrence of inflammation in the human body [23,24,25,26]. Out of these, zinc possesses the most suitable degradation rate [27,28], and, as an essential trace element in the human body, it is integral for nucleic acid metabolism and in the induction of bone cell growth [29,30,31]. Some parameters of various porous materials are summed up in Table 1. Several fabrication methods for porous zinc have been investigated, including infiltration casting [32], additive manufacturing [33], and selective laser melting [33]. Compared with these technologies, the protein foaming method is one of the most promising approaches due to its easy attainability, friendly interaction with the environment, and harmless effect on the human body [34].

Various cell structures for porous ceramics and metals can be produced by means of the protein foaming method [35]. Pore structures rely on the diversity of amino acids of proteins to determine the good binder properties of proteins [36,37,38,39]. In particular, forming a steady two-dimensional network structure with a protein molecule would cause the bubbles obtained through mechanical stirring in a slurry to be stable [35,40]. Currently, the protein foaming method is adopted to produce porous materials of Ti_4_Al_6_V [41] and porous ceramic [42], which cannot degrade within the human body. Porous zinc, however, is promising for use as a substitution for cancellous bone.

In this work, various proteins were used as foaming agents to prepare porous zinc [43,44]. The pore structures and microstructures were characterized, and the compressive properties and corrosion behaviors in simulated body fluid (SBF) were also investigated.

## 2. Materials and Methods

### 2.1. Raw Materials

**Figure 1:**
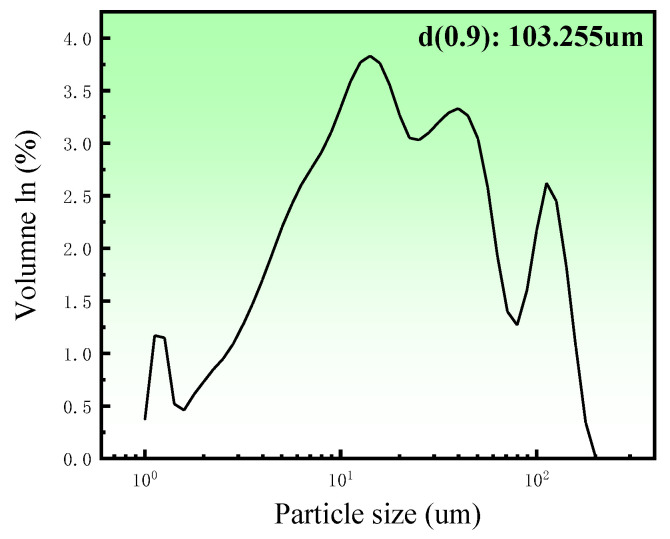
Zinc particle size distribution.

Porous zinc was produced from zinc powder (granularity: 103 μm; density of zinc powder; 7.14 g/cm^3^; 99.99%), egg white protein (EWP) purchased from a local market, bovine bone collagen protein (BBCP; Shaanxi Chenming biological Co., Ltd., Xi’an, China), and fish bone collagen protein (FBCP; Dongsheng Biotechnology Co., Ltd., Guangzhou, China). Sucrose (AR) and polyvinyl alcohol (AR) were added as a binder and a dispersant. The distribution of particle sizes for zinc powder is illustrated in Figure 1; it was measured by using a laser particle size analyzer (Master Size 2000, Malvern Panalytical, Malvern, UK).

### 2.2. Fabrication of Porous Zinc

**Figure 2:**
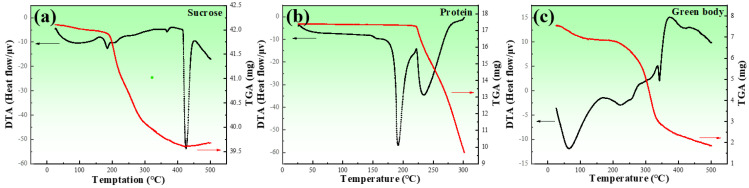
DTA and TGA for the raw materials: (**a**) sucrose; (**b**) proteins; (**c**) green body of porous zinc. The red line represents the TGA, and the black line represents the DTA.

In order to determine the decomposition temperatures of sucrose, proteins, and a mixture of zinc powder and additives, a sintering process, differential thermal analysis (DTA, NETZSCH Gerätebau GmbH, Selb, Germany), and thermogravimetric analysis (TGA, NETZSCH Gerätebau GmbH, Selb, Germany) were used, as shown in Figure 2. of the endothermic and exothermic peaks of the three types of proteins used in this study almost overlapped; Figure 2b represent all of the proteins used in this study.

Figure 2a shows the result of the DTA and TGA for sucrose, which exhibited endothermic peaks at 190 and 236 °C, respectively. The endothermic peak in DTA was attributed to the phase transition that occurred during heating. The sudden drop in the TGA was due to the gases released during thermal decomposition. In combination with the sudden drop in the TGA curve at around 213 °C, this indicated the melting and decomposition of sucrose. Figure 2b presents the results of the DTA and TGA for the proteins, where three endothermic peaks can be observed, and the mass loss appeared from the beginning of the heating. It was obvious that the proteins were decomposing. The decomposition rate increased from about 200 °C. Figure 2c exhibits the results of the DTA and TGA for the mixture. The TGA curve sharply decreased at about 200 °C. Four endothermic peaks at around 188, 207, 373, and 425 °C could be observed, corresponding to the melting and decomposition of sucrose, the decomposition of the proteins, and the melting of the zinc powder, respectively.

**Figure 3:**
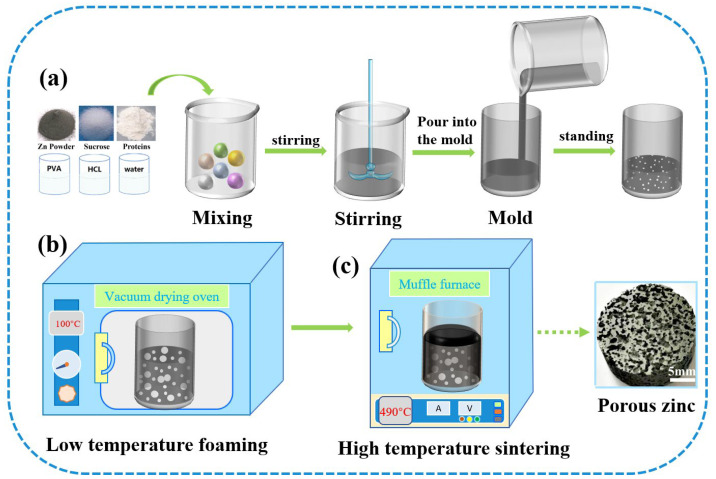
Fabrication process of porous zinc.

Figure 3 shows the fabrication process of porous zinc, which could be divided into three stages. (a) The slurry preparation stage: The zinc powder, protein (EWP, BBCP, or FBCP), sucrose, polyvinyl alcohol, and dilute HCl were added into deionized water. Table 2 shows the composition and content of the slurry. By carrying out substantial experiments, optimal parameters were obtained and porous zinc was successfully fabricated. Proteins were chosen according to the relevant literature [43,44]. A stirring time of 5–20 min at a speed of 80–100 rpm was necessary to introduce numerous air bubbles. The slurry was then allowed to stand at room temperature for 0–20 min. (b) The low-temperature foaming stage: The slurry was placed in a drying oven for foaming at a temperature of 70–100 °C for a period of 2–4 h, during which the moisture was removed and a preliminary porous structure formed. It can be inferred from Figure 2 that, at this temperature, the proteins and sucrose did not decompose. (c) High-temperature sintering stage: A preform covered with graphite was sintered at a temperature of 435–490 °C for 7–10 h in order to prevent the samples from cracking. The samples were then taken out to cool down when the sintering was completed. It can be observed from Figure 2 that the proteins and sucrose decomposed at this temperature and released gases, which affected the morphology of the porous zinc.

### 2.3. Property Characterization

The porosity of the porous zinc was measured with the following equation.

(1) $$P=(1-{\frac{\rho }{\rho^{s}}}^{*})\;\times \;100\%$$

where ρ* and *ρ_s_* are the densities of the porous zinc and the cell wall material, respectively.

The pore size was measured by using Image J on SEM images, and the distribution of pore sizes was obtained by using statistics. Field-emission scanning electron microscopy (Quanta250FEG, FEI Co., Ltd., Hillsboro, OR, USA) was used to observe the pore morphology, pore size, and distribution. X-ray diffraction patterns of porous zinc were obtained using an X-ray diffractometer (D8 ADVANCE, Bruker-AXS Co., Ltd., Karlsruhe, Germany) with Cu–Kα radiation at 40 kV with a scanning rate of 10°/min in the range of 10–90°.

Quasi-static compression tests were conducted in a CMT5105 material testing system (MTS Co., Ltd., Suncheon-si, Korea) with a strain rate of 10^−3^ on specimens with dimensions of 15 mm × 18 mm [45]. At least five tests were conducted for each specimen to guarantee the reliability of the results.

The corrosion behavior of porous zinc produced with FBCP was investigated with an in vitro immersion test in SBF [46] for up to 32 d based on ASTM G31-12. The chemical composition of the solution is indicated in Table 3. The pH of the SBF solution was between 7.4 and 7.45 and was adjusted with 1 M HCl. The diameter and height of the specimen for immersion are 10 mm and 10 mm respectively. The volume of SBF added was 20 mL/cm^2^ [47] according to the surface area of the porous zinc, supposing that it was made up of many spheres. The size and number of spheres were related to the porosity and average pore size of porous zinc. The surface areas (cm^2^) of the samples were calculated with Equation (2).

(2) $$A=4\;\pi \;\left(\frac{d^{2}}{2}\right)\times \left(\frac{V\;\times \;P}{\frac{4}{3}\times \pi \;{\left(\frac{d}{2}\right)}^{3}}\right)=\frac{6\;V\;P}{d}$$

where *d* is the average diameter (cm), *V* is the volume of the samples (cm^3^), and *P* is the porosity of the samples (%).

The immersion temperature was 37 ± 0.5 °C, and the solution was renewed every two days. During immersion, the amounts of zinc ions in the SBF were evaluated with a spectrophotometer (722N, Shang Hai Jing Hua Instrument, Shanghai, China). The degradation byproducts of the porous zinc were removed according to the method [48] using chromic acid solution (200 g/L CrO_3_). The process of cleaning the degradation byproducts was carried out at 80 °C for 1 min, and then alcohol and water were used to clean again at room temperature. The corrosion rate (mm/year) was calculated with the following equation [47].

(3) $$\mathrm{CR}=8.76\;\times \;10^{4}\;\frac{M}{A\;t\;\rho }$$

where *M* is the mass loss weight (g), *A* is the surface area of the samples (cm^2^), *t* is the test duration (h), and *ρ* is the density of zinc (g/cm^3^).

Field-emission scanning electron microscopy (Quanta250FEG, FEI Co., Ltd., Hillsboro, America) was used to observe the pore morphology and corrosion products of porous Zn. X-ray diffraction patterns of porous zinc were obtained using an X-ray diffractometer with Cu–Kα radiation at 40 KV with a scanning rate of 10°/min in the range of 10–90°.

## 3. Results and Discussion

### 3.1. Pore Structure of Porous Zinc

**Figure 4:**
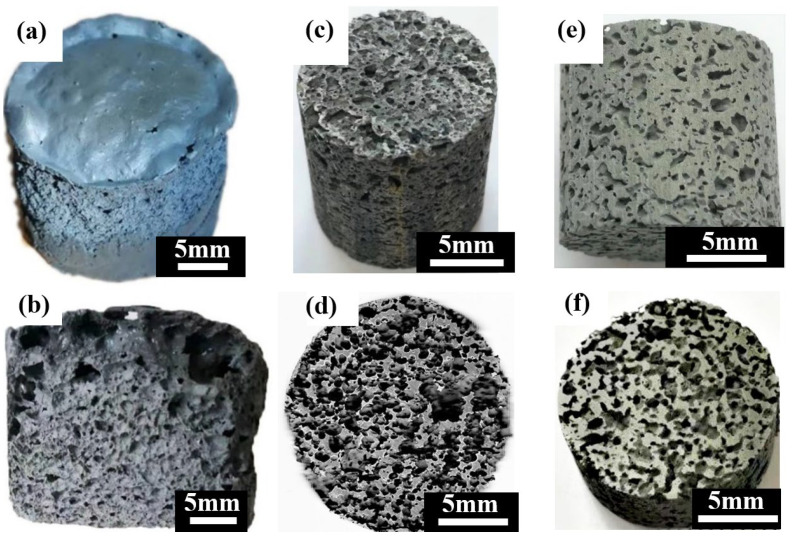
Macrostructures of porous zinc using different proteins: (**a**,**b**) EWP; (**c**,**d**) BBCP; (**e**,**f**) FBCP.

Figure 4 shows the macrostructures of the porous zinc prepared by using different proteins. The samples prepared with EWP (Figure 4a,b), BBCP (Figure 4c,d), and FBCP (Figure 4e,f) exhibited similar structures.

The porosity of porous zinc increased if the stirring time was longer and more protein and sucrose were added. A maximum porosity of nearly 85% was achieved with a longer stirring time and more sucrose and protein. A minimum porosity of nearly 50% was achieved with shorter agitation time and less protein and sucrose. The porosity could even be further decreased below the minimum value by further reducing the stirring time and the content of protein and sucrose.

**Figure 5:**
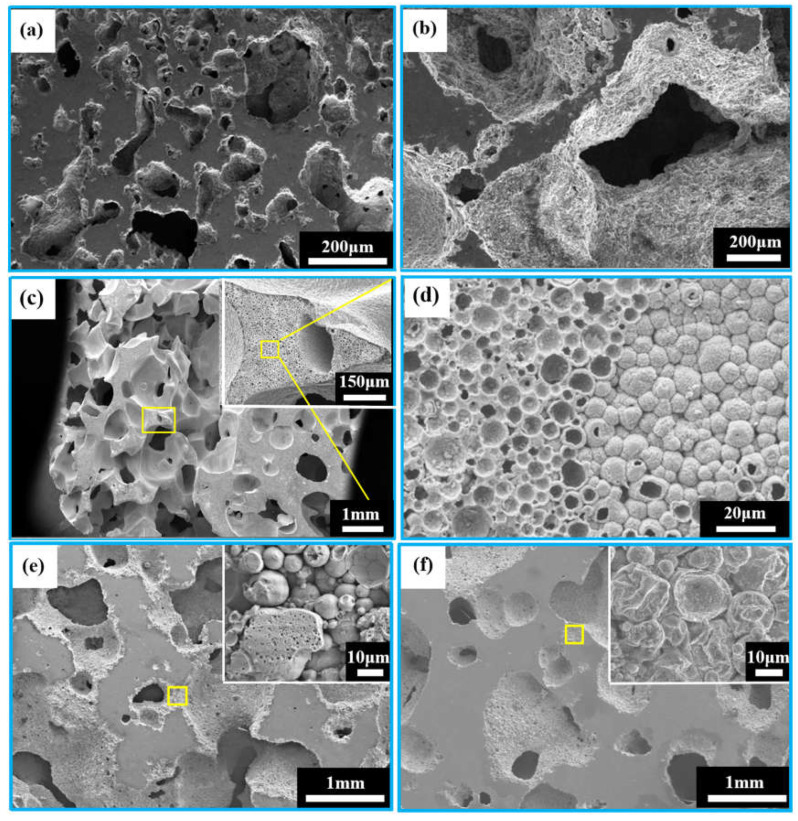
SEM images of porous zinc using different proteins: (**a**,**b**) EWP with a porosity of 76% ± 2%; (**c**,**d**) BBCP with a porosity of 52% ± 2% (stirring time: 20 min, sintering temperature: 490 °C); (**e**,**f**) FBCP with a porosity of 53% ± 2%.

Figure 5a–f show the microstructures of porous zinc produced with different proteins. An interconnected open-cell structure could be observed in three samples. The pores of the porous zinc foamed with EWP (Figure 5a,b) with a porosity of 76% ± 2% were irregular and dense. However, the sample foamed with BBCP (Figure 5c,d) with a porosity of 76% ± 2% showed circular and regular pores. The pore walls contained numerous hollow spheres (Figure 5d). These could have been contributed by the gas produced through the decomposition of the organic additives during sintering, which was then wrapped by the partly molten zinc. Compared to the samples foamed with EWP and BBCP, that foamed with FBCP with a porosity of 53% ± 2% displayed a larger pore size and thicker pore walls, as shown in Figure 5e,f. A large number of small and roughly circular pores could be found. The results suggested that the porous zinc produced with BBCP/FBCP had a suitable pore structure for implants.

**Figure 6:**
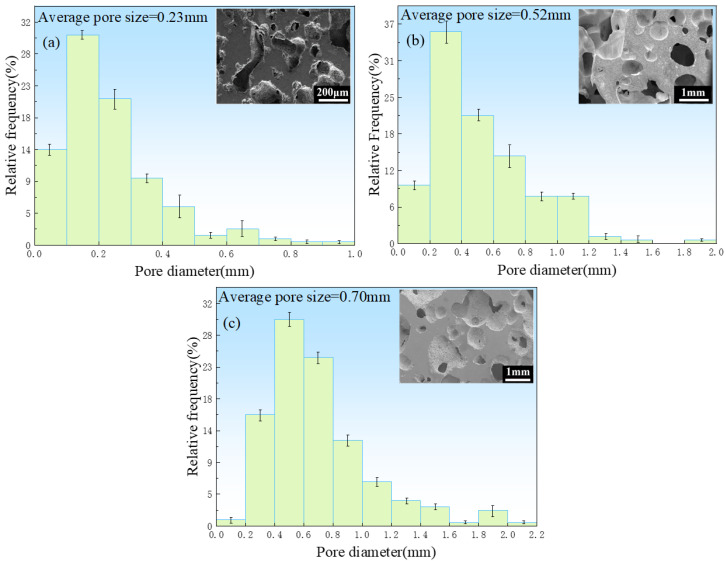
Diameter distribution in porous zinc produced with (**a**) EWP with a porosity of 76% ± 2%, (**b**) BBCP with a porosity of 52% ± 2%, and (**c**) FBCP with a porosity of 53% ± 2%.

**Figure 7:**
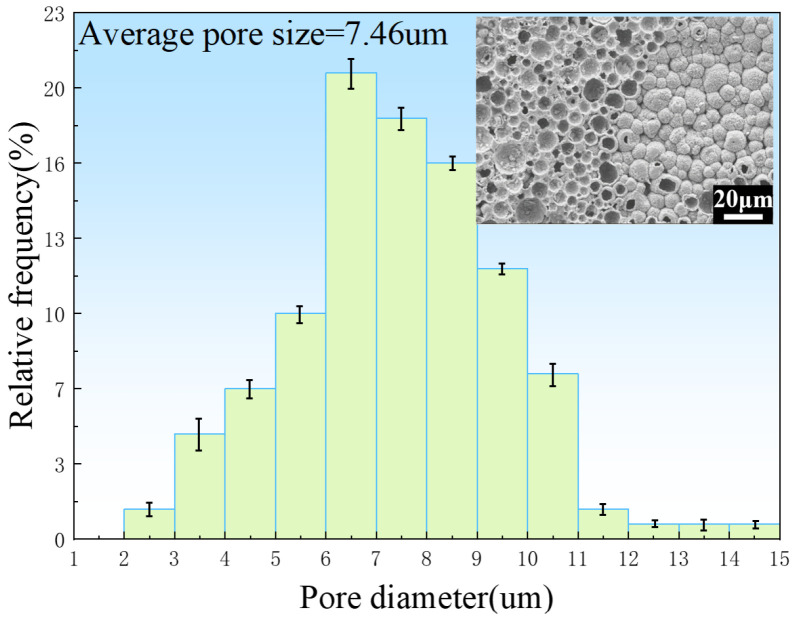
Size distribution of micropores in the pore walls of the porous zinc produced with BBCP with a porosity of 52% ± 2 %.

Figure 6 shows the pore size distributions of the porous zinc prepared with EWP, BBCP, and FBCP; the porosity of the various samples of porous Zn was 76% ± 2%, 52% ± 2%, and 53% ± 2%, respectively. It can be seen that the pore sizes of the samples foamed with BBCP and FBCP were much larger than that of the sample foamed with EWP. Figure 7 shows the distribution of pore sizes of the micropores in the pore walls of the sample produced with BBCP that was shown in Figure 5c. The average diameter of the hollow spheres was 7–8 μm. It could be observed that the porous zinc produced with FBCP possessed the highest average pore size, making it more suitable as an implant for the growth of bone tissue.

**Figure 8:**
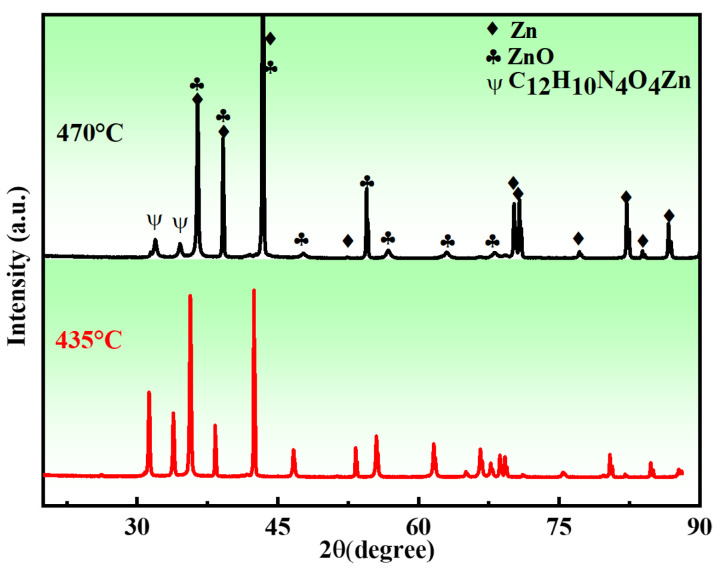
XRD patterns of the porous zinc produced with FBCP with a porosity of 53% ± 2% at different sintering temperatures: (**top**) 470 and (**bottom**) 435 °C.

The XRD patterns of the porous zinc samples sintered at different temperatures are shown in Figure 8. It can be seen that the sintered samples were mainly composed of Zn, ZnO, and C_12_H_10_N_4_O_4_Zn. The reason for the formation of C_12_H_10_N_4_O_4_Zn can be attributed to the combination reaction that occurred during the decomposition of proteins and sucrose and the melting of zinc. The peak intensity of C_12_H_10_N_4_O_4_Zn decreased with increasing sintering temperature, demonstrating that a higher sintering temperature was beneficial for the removal of organics.

### 3.2. Compression Properties

**Figure 9:**
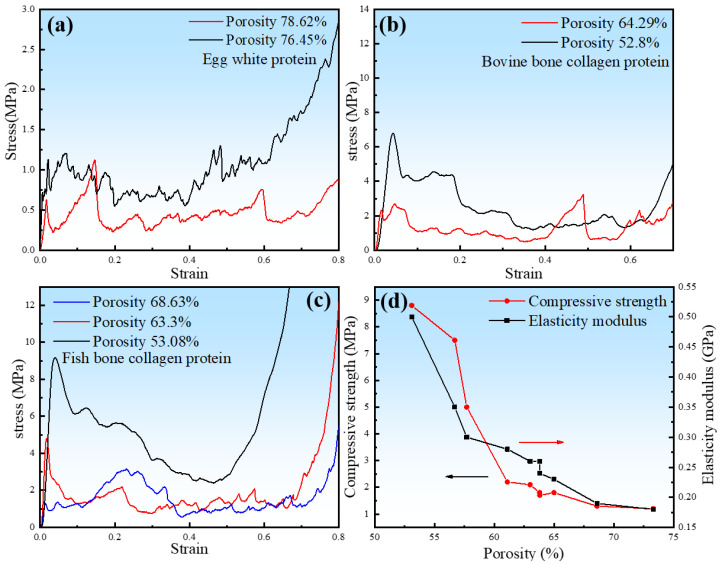
The effect of porosity on compression stress–strain curves and the relationship of porosity with the compressive strength and modulus of elasticity: (**a**) EWP, (**b**) BBCP, and (**c**) FBCP. (**d**) The relationship of porosity with compressive strength and the modulus of elasticity in porous zinc produced with FBCP.

The compressive stress–strain curves of porous zinc are shown in Figure 9. The curves could be divided into three typical stages. The first was the elastic stage. The FBCP samples showed a higher peak stress and elastic modulus compared to those of the BBCP and EWP samples. The EWP samples showed the lowest peak stress and elastic modulus. The second was the stress fluctuation stage, during which the pore walls collapsed with increasing stress. A large stress drop occurred at this stage. It is worth noting that the stress plateau decreased with strain at the second stage when the porosity of sample was lower than 60%, which can be attributed to the brittle fracture of the pore walls. Finally, a densification stage was exhibited, in which the subtended pore walls came into contact, causing an abrupt increase in stress.

The porous zinc produced with EWP with porosities of 78.62% and 76.45% presented the lowest compressive strengths of 0.63 and 0.72 MPa (Figure 9a). The BBCP porous zinc samples with porosities of 62.29% and 52.82% presented the compressive strengths of 2.33 and 6.80 MPa (Figure 9b). Figure 9c presents the stress–strain curves of the porous zinc produced with FBCP with porosities of 68.63%, 63.30%, and 53.08%. The corresponding compressive strengths were 1.30, 4.8, and 9.2 MPa, respectively. The fabricated porous zinc produced with FBCP/BBCP/EWP exhibited porosities and mechanical properties close to those of a human cancellous bone, with porosities from 30% to 95%, a compressive strength from 2 to 12 MPa, and a modulus of elasticity from 0.1 to 5 GPa [24]. The results exhibited that the porous zinc produced with FBCP (porosity of 53.08%) had the highest compressive strength compared to the porous zinc produced with EWP/BBCP. As described above, the porous zinc produced with FBCP possessed the highest compressive properties and a suitable pore structure, which was what we needed.

The effect of porosity on the compressive strength and elastic modulus of porous zinc presented in Figure 9d indicated that the compressive strength and elastic modulus of porous zinc decreased with the porosity.

### 3.3. Corrosion Behavior

**Figure 10:**
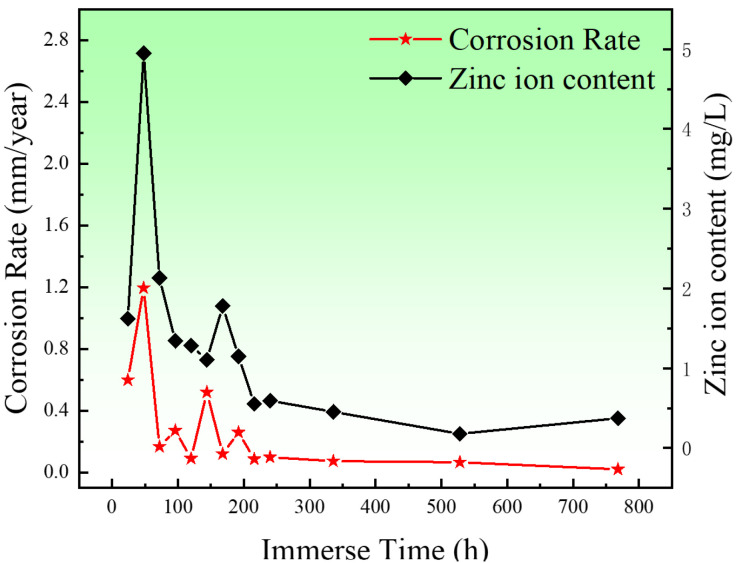
Corrosion rates of the porous zinc prepared with FBCP with a porosity of 53–58% and the content of zinc ions.

Figure 10 shows the corrosion rates and content of Zn^2+^ of the porous zinc produced with FBCP with a porosity of 53–58%. The corrosion rates of the porous zinc decreased with the increase in immersion time, indicating that the formation of corrosion products had a protective effect on the surface of the porous zinc. In two days, a quick improvement in the corrosion rate and content of Zn^2+^ was observed because bare porous zinc was in contact with the SBF solution. The occurrence of a sudden decrease in the corrosion rate on the third day was mainly caused by the production of corrosion products on the surface, which impeded the corrosion process. As the pore structure was not absolutely the same for the experiments, the corrosion rate of the porous zinc underwent minor fluctuations. Moreover, with the increase in the corrosion products, the corrosion rate decreased, and consequently, the corrosion rate was close to 0.02 mm/year [16], which satisfied the requirements for corrosion rates in bone implants of less than 0.5 mm/year [16], indicating that porous zinc could be applied for cancellous bone implantations.

**Figure 11:**
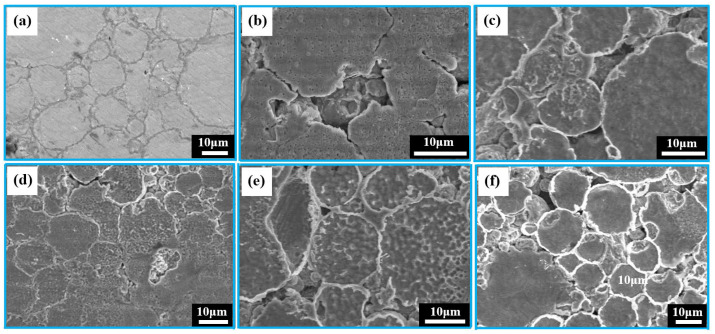
SEM images of porous zinc produced with FBCP with a porosity of 53–58% after immersion for different periods of time: (**a**) 0, (**b**) 5, (**c**) 10, (**d**) 15, (**e**) 25, and (**f**) 32 d.

The corrosion behavior of the porous zinc produced with FBCP was evaluated through the characterization of the SEM surface morphologies of the degraded porous zinc after 32 days of immersion, as shown in Figure 11a–f. The pore sizes of the porous zinc (Figure 11a–f) produced with FBCP ranged from 0.01 to 2.08 mm, with a porosity of 53–58%. Figure 11 shows that the majority of the corrosion occurred in the bonding of particles, and corrosion pits occurred on the surfaces of the particles. It appeared that the bonding of the particles was corroded more easily than their surfaces. This may be attributed to the oxidation and cathodic reactions.

**Figure 12:**
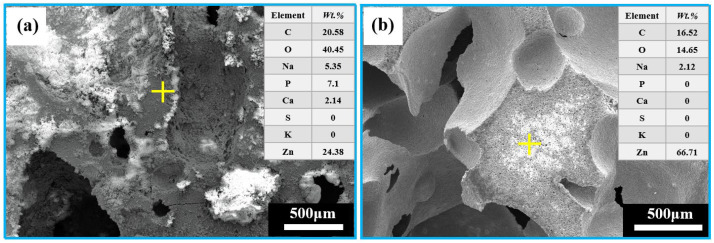
EDS analysis of the porous zinc produced with FBCP after immersion: (**a**) porous zinc produced with FBCP covered with corrosion products before cleaning; (**b**) porous zinc produced with FBCP after cleaning.

It can be observed from Figure 12 that the EDS analysis after immersion of the porous zinc produced with FBCP indicated that the surface covered with corrosion products consisted of C, O, Zn, Na, P, Ca, S, and K (Figure 12a), while the surface after cleaning consisted of C, O, Zn, and Na (Figure 12b), indicating that the corrosion product mainly consisted of P, Ca, S, and K, and that P-based and Ca-based corrosion products were located on the top of the corrosion layer.

**Figure 13:**
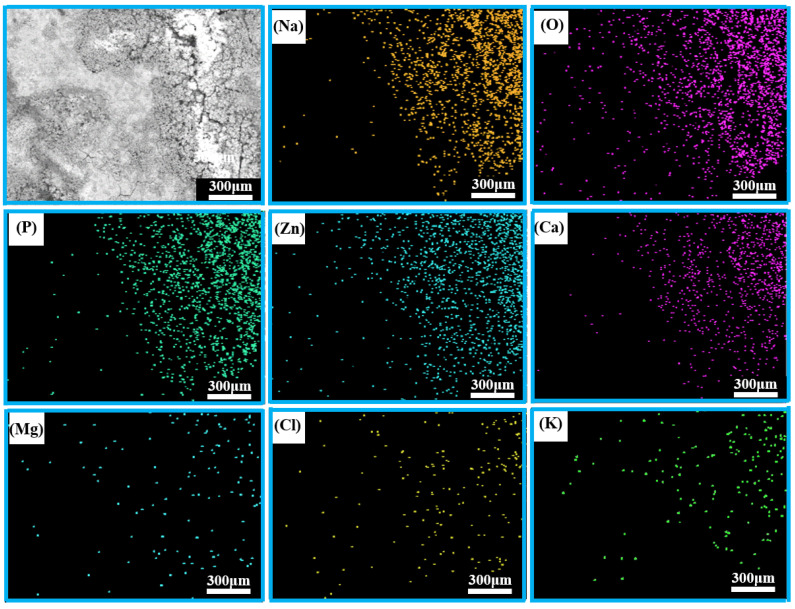
EDS spectra of the porous zinc produced with FBCP after immersion.

The corresponding EDS mapping results for the porous zinc produced with FBCP covered with the corrosion product are shown in Figure 13; the whole region mainly contained Na, O, P, Zn, Ca, Mg, Cl, and K. Many corrosion products were distributed on the surface of the porous zinc. There were more corrosion products attached to the surface in the bright portion with micro-cracks.

**Figure 14:**
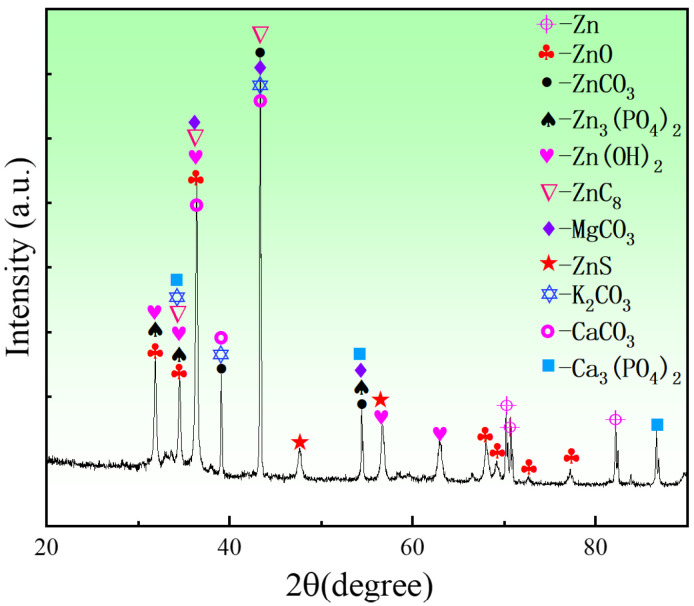
XRD patterns of porous zinc produced with FBCP after immersion in SBF for 32 d.

To analyze the phase compositions of the corrosion products of the porous zinc, EDS and XRD (Figure 12, Figure 13 and Figure 14) were performed; the corrosion morphology was also analyzed (Figure 11, Figure 12 and Figure 13). The XRD patterns of the corroded porous zinc produced with FBCP after immersion in SBF for 32 d are given in Figure 14. The corrosion products of porous zinc may include ZnO, ZnCO_3_, Zn_3_(PO_4_)_2_, Zn(OH)_2_, CaCO_3_, Ca_3_(PO_4_)_2_, and Ca_3_Zn_2_(PO_4_)_2_CO_3_(OH)_2_ [31,49,50,51,52], which can be degraded in the human body through metabolism, and they have no toxicity. These corrosion products were in agreement with the loose and porous surface morphology of the porous zinc after corrosion (Figure 11, Figure 12 and Figure 13). A passive film, such as Zn_3_(PO_4_)_2_, can protect the underlying porous zinc and delay degradation. However, ZnO enhanced the degradation by forming a galvanic couple with Zn. The degradation of zinc in the nearly neutral environment in the SBF can be described as follows [53].

(4) $$\mathrm{Zn}\;\to {\mathrm{Zn}}^{2+}+2e^{-}$$

(5) $$O_{2}+2H_{2}O+4e^{-}\to 4{\mathrm{OH}}^{-}$$

(6) $${\mathrm{Zn}}^{2+}+2{\mathrm{OH}}^{-}\to \mathrm{Zn}{\left(\mathrm{OH}\right)}_{2}$$

(7) $$2{\mathrm{Zn}}^{2+}+2{\mathrm{OH}}^{-}\to 2\mathrm{ZnO}+H_{2}$$

Zinc hydroxide (Zn(OH)_2_) can redissolve due to chloride attack in physiological conditions; thus, zincite (ZnO) was the more dominant corrosion product, as it had more thermodynamically stable oxidation, and the released Zn ions could react with phosphate ions to form insoluble Zn phosphate (Zn_3_(PO4)_2_) [30].

(8) $$\mathrm{Zn}{\left(\mathrm{OH}\right)}_{2}+2{\mathrm{Cl}}^{-}\to {\mathrm{Zn}}^{2+}+2{\mathrm{OH}}^{-}+2{\mathrm{Cl}}^{-}$$

(9) $$3{\mathrm{Zn}}^{2+}+2{\mathrm{HPO}}_{4}{}^{2-}+2{\mathrm{OH}}^{-}+2H_{2}O\to {\mathrm{Zn}}_{3}{\left({\mathrm{PO}}_{4}\right)}_{2}\cdot 4H_{2}O$$

## 4. Conclusions

In the present study, a new protein foaming–consolidation porous zinc was successfully fabricated with three proteins as consolidating and foaming agents. The effects of stirring time, content of protein and sucrose, and sintering temperature on the microstructure, compressive properties, and corrosion properties of porous zinc were investigated. The main conclusions drawn from the current work are as follows:

1. Porous zinc was produced using three simple steps of slurry preparation, low-temperature foaming, and high-temperature sintering. The processing method is applicable to the preparation of porous zinc with porosities in the range of 50–85.8% and pore sizes in the range of 0.012 to 2.08 mm. The porous zinc produced with FBCP exhibited the highest compressive strength and elastic modulus.
2. The macrostructure of porous zinc can be changed with different protein types, the content of protein and sucrose, the stirring time, and the sintering temperature.
3. The porosity increased with the stirring time, as well as the content of protein and sucrose. The porous zinc produced with FBCP exhibited more circular and regular pores and the largest pore size.
4. The compressive properties of porous zinc were highly dependent on the porosity and types of proteins. Porosity and compressive strength were inversely proportional. Porous zinc prepared with FBCP exhibited a superior compressive strength and elastic modulus. The compressive strength of the porous zinc produced with FBCP was about eight times higher than that of the porous zinc produced with EWP.
5. The main corrosion mechanisms of porous zinc showed that Zn ions would react with hydroxyl ions, carbonate ions, phosphate ions, etc. The corrosion products were determined to be ZnO, ZnCO_3_, Zn_3_(PO_4_)_2_, Zn(OH)_2_, CaCO_3_, Ca_3_(PO_4_)_2_, and Ca_3_Zn_2_(PO_4_)_2_CO_3_(OH)_2_, which can be degraded in the human body through metabolism. The corrosion rate of porous zinc obtained through an in vitro immersion test using simulated body fluid on the thirty-second day was close to 0.02 mm/year.
6. Overall, porous zinc shows an optimal combination of compressive and corrosion properties and is considered as highly promising for the requirements of cancellous bone implantation.

## Figures and Tables

**Table 1:** Performance parameters of porous materials.

Material and Method	Compressive Strength/MPa	Application	Porosity	Ref.
Fe/3DP	16.7	Bone tissue engineering	80~80.6%	[24]
Fe/PU	0.382 ± 0.024	Bone tissue engineering	96~97%	[25]
Fe/TAED	3.5	Tissue engineering	>90%	[26]
Mg/FDHP	11.1~30.3	Bone substitute applications	33~54%	[18]
Mg/PM	4.4~38	Orthopedic applications	12~38%	[21]
Zn/AMC	6~11	Orthopedic applications	22~65%	[27]
Zn/AM	10.8~13.9	Bone substitution	60~67%	[28]
(PF/HAP) PS	0.3~1.1	Bone tissue engineering	79~89%	[11]
Glass-ceramic/PF	2.6~6.2	Biomaterials scaffold	68~78%	[9]
Polymer/3DP	2.6~6.2	Engineering architected foams	68~78%	[10]
Zn/Protein foaming	1.19~9.20	Cancellous bone substitution	50~85.8%	This work

**Table 2:** Slurries consisting of different compositions and contents for the fabrication of porous zinc.

Types of Protein	Content (wt.%)				
	Protein	Sucrose	Polyvinyl Alcohol	Deionized Water	1M HCL
FBCP	0.24	5	5	9.53	0
FBCP	1.2	10	0	8.8	0.67
BBCP	0.24	15	3	8.96	0.45
BBCP	0.80	5	0	3.9	0.22
EWP	3	5	5	4.7	0
EWP	15	10	3	2.92	0.67

**Table 3:** Regents of preparing SBF.

Rank	Reagent	Content	Purity	Molecular Weight
1	NaCl	8.035 g/L	99.5%	58.4430 g/mol
2	NaHCO_3_	0.355 g/L	99.5%	84.0068 g/mol
3	KCl	0.225 g/L	99.5%	74.5515 g/mol
4	K_2_HPO_4_ 3H_2_O	0.231 g/L	99.0%	228.2220 g/mol
5	MgCl_2_ 6H_2_O	0.311 g/L	99.0%	203.3034 g/mol
6	1.0M HCl	39 mL/L	-	-
7	CaCl_2_	0.292 g/L	95.0%	110.9848 g/mol
8	NaSO_4_	0.072 g/L	99.0%	142.0428 g/mol
9	Tris	6.118 g/L	99.0%	121.1356 g/mol
10	1.0M HCl	0~5 mL/L	-	-

## Data Availability

Not applicable.
